# Lethal influenza virus A H1N1 infection in two relatives with autosomal dominant GATA-2 deficiency

**DOI:** 10.1186/cc11953

**Published:** 2013-03-19

**Authors:** J Sole-Violan, I Sologuren, E Betancor, S Zhang, C Pérez, E Herrera-Ramos, M Martínez-Saavedra, M López-Rodríguez, J Pestano, J Ruiz-Hernández, J Ferrer, F Rodríguez de Castro, J Casanova, C Rodríguez-Gallego

**Affiliations:** 1 Hospital GC Dr Negrín, Las Palmas de GC, Spain; 2 Universidad Las Palmas de GC, Spain; 3 The Rockefeller University, New York, NY, USA

## Introduction

Most individuals infected with the 2009 pandemic H1N1 influenza A virus (IAV) (H1N1pdm) experienced uncomplicated flu. However, in a small subset of patients the infection rapidly progressed to primary viral pneumonia (PVP) and a minority of them developed ARDS. Inherited and acquired variability in host immune responses may influence susceptibility and outcome of IAV infection. However, the molecular nature of such human factors remains largely elusive.

## Methods

We report three adult relatives with the autosomal dominant GATA-2 deficiency. P1 and his son P2 had a history of myelodysplastic syndrome and a few episodes of mild respiratory infections. They developed PVP by H1N1pdm which rapidly evolved to ARDS. They died at the age of 54 and 31, respectively.

## Results

Patients were heterozygous for a novel R396L mutation in GATA2. Like other patients with GATA-2 deficiency, the three relatives had absence of peripheral NK and B cells and monocytopenia. However a high number of plasma cells, which were found to be pauciclonal, were observed in peripheral blood from P1 during H1N1pdm infection. P1 and P2 had normal levels of immunoglobulins and IgG antibodies against common viruses. Microneutralization test showed that P1 produced normal titers of neutralizing antibodies against H1N1pdm and against the previous annual H1N1 strain. Our results suggest that a few clones of long-living memory B cells against IAV expanded in P1; and that these cells produced cross-reactive antibodies against H1N1pdm, similar to those recently described. During the flu episode P1 had a strong increase of IFNγ-producing T cells and of IFNγ production. The Th1-related chemokines CXCL10 and CXCL9, as well as IFNγ, MCP-1 and IL-8, were strongly elevated in serum from P1 and P2 in the course of H1N1pdm infection.

## Conclusion

GATA-2 deficiency is the first described Mendelian inborn error of immunity underlying severe IAV infection. Primary immunodeficiencies predisposing to severe IAV infections may debut, even in adults without a history of previous severe infections. The massive IFNγ-mediated cytokine storm may explain the fatal course of H1N1pdm infection in our patients.

